# Hybrid Artificial Root Foraging Optimizer Based Multilevel Threshold for Image Segmentation

**DOI:** 10.1155/2016/1476838

**Published:** 2016-09-20

**Authors:** Yang Liu, Junfei Liu, Liwei Tian, Lianbo Ma

**Affiliations:** ^1^Peking University, Beijing 100871, China; ^2^Shenyang University, Shenyang 110044, China; ^3^Northeastern University, Shenyang 110318, China

## Abstract

This paper proposes a new plant-inspired optimization algorithm for multilevel threshold image segmentation, namely, hybrid artificial root foraging optimizer (HARFO), which essentially mimics the iterative root foraging behaviors. In this algorithm the new growth operators of branching, regrowing, and shrinkage are initially designed to optimize continuous space search by combining root-to-root communication and coevolution mechanism. With the auxin-regulated scheme, various root growth operators are guided systematically. With root-to-root communication, individuals exchange information in different efficient topologies, which essentially improve the exploration ability. With coevolution mechanism, the hierarchical spatial population driven by evolutionary pressure of multiple subpopulations is structured, which ensure that the diversity of root population is well maintained. The comparative results on a suit of benchmarks show the superiority of the proposed algorithm. Finally, the proposed HARFO algorithm is applied to handle the complex image segmentation problem based on multilevel threshold. Computational results of this approach on a set of tested images show the outperformance of the proposed algorithm in terms of optimization accuracy computation efficiency.

## 1. Introduction

Image segmentation is an important image preprocessing technique with primitive operations for image recognition [1, 2]. The goal of image segmentation is to partition an original image into a suit of disjoint sections or regions by gray values and texture structures [3]. Generally, there is a strong correlation between the objects of these disjoint regions in the image. Bithreshold or multilevel threshold based segmentation methods have been deeply developed and employed in various practical applications. The key issue to this segmentation method is the computational determination of the involved threshold. A broad variety of threshold based segmentation methods have been proposed, including conventional approaches [4] and intelligent approaches [5, 6]. Among them, the classical Otsu criterion shows significant merits of simplicity and high efficiency, which determines the appropriate thresholds according to intrinsic profile characteristic of histogram [7]. As a matter of fact, the Otsu transforms the multilevel threshold segmentation into an optimization problem, which tends to maximize intercluster variance of subpartition. However, due to the exhaustive property of this approach, the computational complexity will rise exponentially with the increasing of the threshold number [8, 9].

Recently, due to their excellent abilities of tackling complex NP-hard problems, metaheuristics such as artificial bee colony [10, 11], particle swarm optimization [12], artificial ant colony [13], differential evolution [14], firefly algorithm [15], wind driven optimization [16], and bacterial foraging algorithm [17] have been adopted widely in threshold image segmentation. It is worth noting that those metaheuristics are generally inspired form intelligent behaviors of animals that have foraging strategies. The survival wisdom of plants, as another typical species of foraging organisms, has received little attention due to their specific lifestyle [18]. However, terrestrial plants have prominent adaptability and sensing ability to use environmental information as a basis for governing their growth orientation and root system development [19]. Logically, such adaptive growth processes can provide novel insights into new computing paradigm for global optimization [20–22]. References [23, 24] have proposed and developed the novel and effective EA variants by using a hybridization of life-cycle and optimal search strategies and obtain significant performance improvement, which shows a novel and effective computation framework for related scientists. How to deliberately design novel evolutionary computation model and algorithm is increasingly becoming an area of active research; taking a promising example, a representative ARFO algorithm is proposed by Ma et al. in [22] and has received a surge of attention [23, 24]. Essentially, the ARFO provides an open and extensible biocomputation framework and model for scientists in the field of optimization theory to exploit new bioinspired algorithms.

Thus, this paper develops a novel hybrid artificial root foraging optimizer (HARFO) which synergizes the idea of coevolution and root-to-root communication strategy. In the proposed model, all roots can be generally divided into the main roots and lateral roots according to the auxin concentration. The main root as the strongest individuals can branch and regrow under effect of hydrotropism. The lateral root involves many branches derived from the main root, and its growth direction orients from corresponding main root [25, 26]. Furthermore, in the root-to-root communication, through different effective topology, individual roots share more information from the elite roots in the early exploration stage of the algorithm. With multipopulation coevolution mechanism, the hierarchical population of roots can be structured with enhanced interactions of individual behaviors from different subpopulations. By incorporating a set of hybrid strategies, the proposed HARFO can be claimed very effective and efficient because the exploitation and exploration can be elaborately balanced, which guarantees finding the optimal thresholds at a more reasonable time.

This paper is structured as follows: In Section 2, a brief overview of the proposed hybrid artificial root foraging optimizer model and algorithm is presented.  Section 3 experimentally compares HARFO with other well-known algorithms on a set of benchmark functions. In Section 4 the implementation of HARFO for multilevel threshold for image segmentation is conducted. In Section 5 final conclusion is outlined.

## 2. Hybrid Artificial Root Foraging Optimizer

### 2.1. Artificial Root Foraging Optimization (ARFO) Model

This section briefly describes the classical ARFO proposed in [22], which simulates the intelligent foraging behaviors of plant roots. As depicted in [22], in order to idealize biological plant root growth behaviors, some criteria are presented as follows.

*Auxin Concentration*

 The root's adaptive growth is conducted by auxin concentration, which significantly influences the information exchange among root tips. The auxin concentration regulates the roots' spatial structure, after new roots germinate and grow, and it is dynamically reallocated instead of static.

*Growth Strategies*

 Regrowing: one root apex elongates forward (or sideways) in the substrate.
 Branching: one root apex produces daughter root apices.

*Root Classification*

 The whole root system generally consists of three categories sorted by the auxin concentration from high to low: the main roots, the lateral roots, and the dead roots

*Root Tropisms*

 The growth trajectory of plant roots is influenced by hydrotropism, which makes the growing direction of the root tips towards the optimal individual position.

Generally, each root implements different growth strategies and operators according to the above criteria. Each main root regrows (i.e., elongates itself) while branching new individuals once some conditions are met. After each growth cycle, some deteriorated roots are selected as the dead roots to be eliminated from current population.

#### 2.1.1. Auxin Regulation

Supposing that *A*_*i*_ as the auxin concentration is used to exhibit the nutrient distribution in artificial soil environment, then it can be stated mathematically as below:

(1) $$\begin{array}{c} f_{i}=\frac{{fitness}_{i}-f_{min}}{f_{max}-f_{min}}. \end{array}$$

Then

(2) $$\begin{array}{c} A_{i}=\frac{f_{i}}{\sum_{j=1}^{S}f_{i}}, \end{array}$$

where fitness_*i*_ is the functional fitness value, *f*_*i*_ is the normalization fitness value of the root *i*, *f*_min_ and *f*_max_ are the maximum and minimum of the current population, respectively, and *S* is the size of current population. In each cycle of root growth process, all root taps are sorted by auxin concentration values defined above. In our model, half of the sorted population are selected as main roots while the rest of roots are identified as lateral roots.

#### 2.1.2. Main Roots' Growth: Regrowing and Branching

According to the growth strategy of main root in criterion for the plant root growth behaviors, a main root with high *A*_*i*_ value has strong growth ability of implementing both regrowing operator and branching operator.

*(i) Regrowing Operator*. In this regrowing process, the strong main root can sense environmental stimuli (i.e., nutrient distribution) and use this information to govern its growth orientation. Then, the formulation of this operator is given as below:

(3) $$\begin{array}{c} x_{i}^{t}=x_{i}^{t-1}+l\cdot rand\cdot \left(\;x_{lbest}-x_{i}^{t-1}\;\right), \end{array}$$

where *x*_*i*_^*t*^ and *x*_*i*_^*t*−1^ are defined as the position of root *i* at time step *t* and *t* − 1, respectively, *l* is a local learning inertia, rand is a random coefficient varying within [0,1], and *x*_*lbest*_ is the local best individual in current population.

*(ii) Branching Operator*. The main roots with higher auxin concentration values have higher probability to branch more individuals. In this operator, for each main root, if its auxin concentration value is more than a branching threshold* T_Branch*, it will start generating a certain number of new individuals as follows:

(4) $$\begin{array}{c} \text{branch}\;\;w_{i}\text{ individuals}\;\text{if }A_{i}>T\_\text{Branch},\\ \text{nobranching}\;\text{otherelse}. \end{array}$$

In principle, the main root in nutrient-rich environment will forage for energy to obtain higher auxin concentration and then produces more branches. Thus, the branch number *w*_*i*_ can be calculated as

(5) $$\begin{array}{c} w_{i}=R_{1}A_{i}\left(\;s_{max}-s_{min}\;\right)+s_{min}, \end{array}$$

where *R*_1_ is a random coefficient within the range [0,1], *A*_*i*_ is the auxin concentration of root *i*, and *S*_max_ and *S*_min_ are the maximal number and minimal number of the new branching individuals, respectively, which are usually preset to 4 and 1, respectively.

The position of a newly branching root is initialized from the parent main root with Gauss distribution *N*(*x*_*i*_^*t*^, *σ* ^2^), where *σ* can be defined as

(6) $$\begin{array}{c} \sigma_{i}={\left(\;\frac{\left(i_{max}-i\right)}{i_{max}}\;\right)}^{n}\cdot \left(\;\sigma_{ini}-\sigma_{fin}\;\right)+\sigma_{fin}, \end{array}$$

where *i* is the current iteration index, *i*_max_ is the maximum of iterations, the initial standard deviation *σ*_ini_ is determined by the range of searching, and *σ*_fin_ donates the final standard deviation.

#### 2.1.3. Lateral Roots Growth: Random Walking

At the *t*th iteration, each lateral root tip generates a random head angle and a random elongation length, given as follows: all lateral roots will conduct random searches at each feeding process; random search strategy is considered to be the most effective foraging strategy in nutrient distributed environment [27, 28]. Each lateral root generates a random growth angle and random elongated length, which is given by

(7) xit=xit−1+rand·lmaxDiφ,φ=δiδiT·δi,

where *l*_max_ is the maximum elongate length unit (i.e., objective function boundary range), rand is a random number with uniform distribution in [0,1], and *φ* is a growth angle computed by a random vector *δ*_*i*_.

#### 2.1.4. Dead Roots' Growth: Shrinkage

In the case that the root does not get enough nutrients from soil, its corresponding auxin concentration is intended to be weak. Once auxin concentration is lower than a certain threshold, the sustained growth probability will be stagnated. This enables the corresponding root to be simply removed from the current population. The branching criterion and dead roots eliminating criterion are listed as follows:

(8) Ni=Ni+wiif  Xi>T_Branch,Ni=Ni−1if  Xi<T_Nmority,

where *N*_*i*_ is the current population size, *T*_*Branch* is the branching threshold, *w*_*i*_ is the branching number defined by (5), and* T_Nmority* is the death threshold.

### 2.2. Root-to-Root Communication

The intrinsic property of the “population” in swarm intelligence is collective intelligence emerging by a number of connected individuals exchanging information in some specific topologies [27–30]. This means that the spatial topological structure plays an important role in enhancing dynamic interaction between individuals and optimizing information propagation path across the structured population.

**Figure 1:**
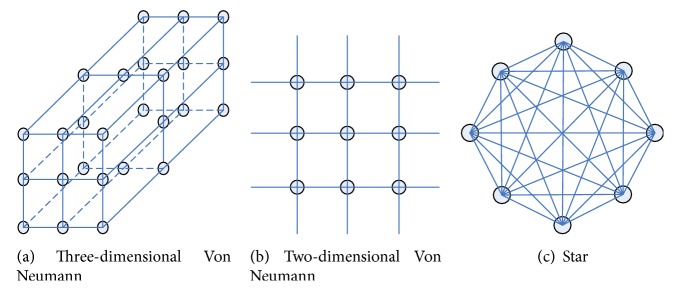
Population topology.

Accordingly, the population topology technique has been strongly recommended for potential improvement of swarm intelligence or evolutionary algorithms [30–33]. Particularly, by lucubrating on the relationship between population topologies structure and algorithmic performances in [29], Kennedy and Mendes conclude that the Von Neumann exhibits better convergence speed on a variety of test functions, as shown Figures 1(a) and 1(b).

In ARFO, (3) shows that an individual's candidate neighborhood termed *x*_*lbest*_ is selected from the entire population, which indicates one central node influences, and is influenced by all other members of the population [26]. In other words, this population topological structure of ARFO essentially falls into the star topology, which is a fully connected neighborhood relation, as shown in Figure 1(c). From [29], it is claimed that the Von Neumann has a lower connectivity while covers a larger search space than the star type, which tends to maintain better diversity of population and reduces the chances of falling into local optima. The procedures of representing Von Neumann structure are listed in Algorithm 1.

### 2.3. Coevolution Mechanism

**Figure 2:**
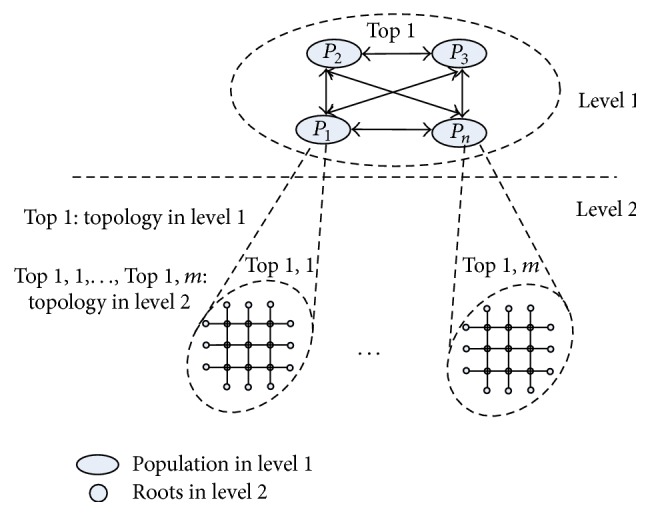
Multispecies coevolution mechanism.

Hierarchy is a common phenomenon in the development of plant root system [25, 26]. With the severity of environmental stress, homogeneous main roots continuously self-grow-branch and evolve while being a part of heterogeneous roots of different plant types and that plant is in turn a part of a specific ecosystem niche [27]. As a result, this hierarchical coevolution approach is incorporated to improve algorithm efficiency via decomposing large-scale problems into simple tasks optimized in parallel. As depicted in Figure 2, the flat ARFO is structured into two levels with different topologies as follows.

Hypothesize that population *P* = {*S*_1_, *S*_2_,…, *S*_*M*_}, and each swarm *S*_*k*_ = {*x*_1_, *x*_2_,…, *x*_*N*_}. In each growth phase, the new individual or agent in level 2 is defined as

(9) xit=xit−1+l1·rand1·xibestt−1−xit−1+l2·rand2·xpbestt−1−xit−1,

where *x*_*ibest*_^*t*−1^ is the best individual within current population which denotes cooperation in level 2 and *x*_*pbest*_^*t*−1^ is the global best individual among all populations which exchanges information across populations in level 1. *l*_1_ and *l*_2_ are the random coefficients. rand_1_ and rand_2_ are random numbers with uniform distribution in [0,1], respectively.

### 2.4. The Proposed Algorithm

**Figure 3:**
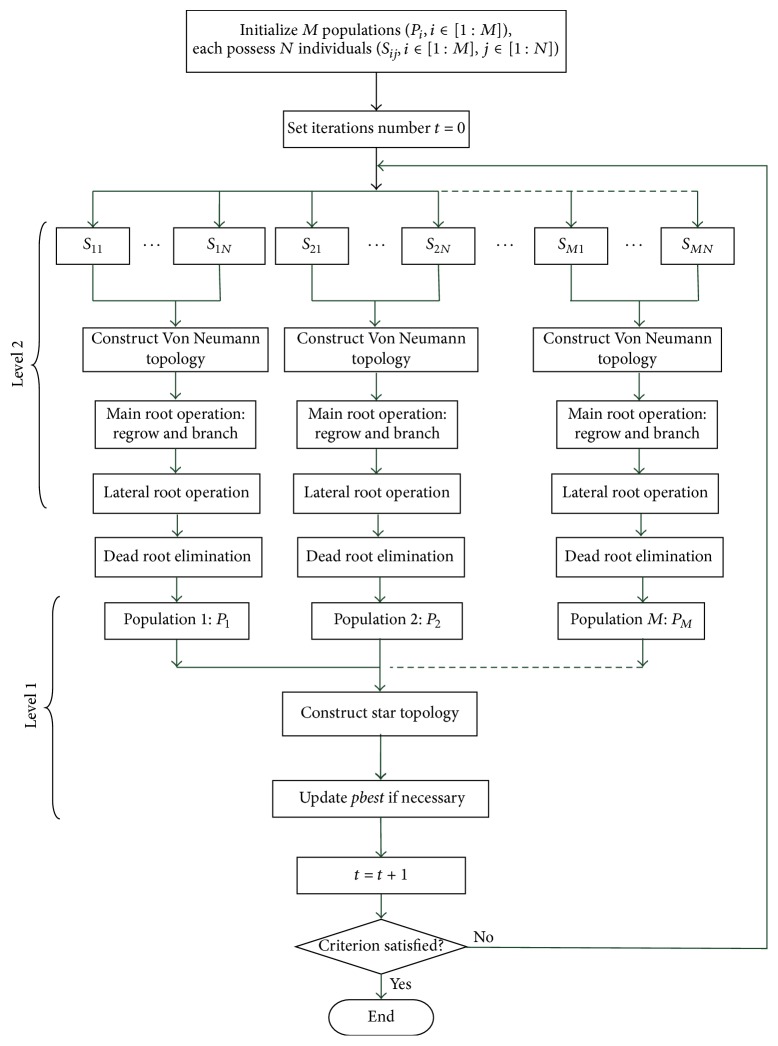
The flowchart of HARFO algorithm.

By hybridizing ARFO with these complex degrees of strategies, namely, root-to-root communication and coevolution mechanism, the hybrid artificial root growth optimizer (HARFO) can regulate the trajectory of each root through the specific topology. Moreover, the evolution of population is guided by historical experience in level 2 and global best information in level 1, which can imply diversity of population. The main procedures of the proposed HARFO are listed in Algorithm 2. The flowchart of HARFO is presented in Figure 3.

## 3. Benchmark Test

### 3.1. Test Functions

For the purpose of performance comparison, the HARFO, together with other state-of-the-art metaheuristic algorithms, is evaluated on a set of test functions from basic benchmarks and CEC 2005 test beds. The definition and mathematical representation of them are available in Table 1 where *f*_1_~*f*_5_ are basic benchmark functions and *f*_6_~*f*_10_ are taken from CEC 2005 test suit, which is complex rotation and shift problem based on the basic test functions.

Furthermore, in order to comprehensively evaluate the performance of the proposed algorithm, a suit of scalable shifted and rotated benchmarks from CEC 2014 test bed is employed in the test [34–36]. The dimensions, initialization ranges, and global optimum of each function (*f*_11_~*f*_20_) are listed in Table 2.

### 3.2. Experimental Configuration

For the purpose of performance comparison, the HARFO is compared with several classical evolutionary algorithms including particle swarm optimization (PSO) [37, 38], cooperative coevolution genetic algorithm (CCGA) [39], pure artificial root foraging optimization algorithm (ARFO) [22], and artificial bee colony algorithm (ABC) [20] on ten test benchmarks given above. Specifically, CCGA is a parallelized GA variant derived from the dimension-distributed coevolution mode, which divides a high-dimensional problem into several lower-dimensional subproblems and then assigns them to corresponding subswarms to coevolve [39]. On each function, the algorithm is independently run 20 times and terminated when the number of function evaluations reaches 100,000 for each run. The common population size associated with ARFO, PSO, and ABC is set to 20.

For PSO, the global version with inertia weight is adopted, and its parameters directly follow the default setting of [37, 38]: the acceleration factors *c*_1_ = *c*_2_ = 2.0 and the decaying inertia weight *ω* starting at 0.9 and ending at 0.4. For ABC, the* limit* is set to *SN* × *D*, where *D* is the dimension of the problem and* SN* is half of population size [20]. For CCGA, the subswarm number is set to 10, and other parameters are the same as its original literature [39]. The parameter setting of HARFO and ARFO can be empirically summarized in Table 3. For the proposed HARFO, the population number *N*, the branching, and dead thresholds should be tuned firstly in next section.

### 3.3. Parameters Sensitivity

*(i) Sensitivity in relation to Population Number M in Level 1*. To scientifically assess the effect of parameter *M*, the following experiment is designed. First, *T*_*Branch* and *T*_*Nmority* are assigned empirically with initial values 10 and 5, respectively. Then *M* is varied from 2 to 17 with a step size 3. For each value of *M*, HARFO is implemented for 20 times on six selected 30-dimensional *f*_11_, *f*_12_, *f*_13_, *f*_14_, and *f*_15_. And computation results in terms of mean and standard deviation are given in Table 4. It can be visibly observed from Table 4 that algorithm with *M* equal to 2 and 8 can perform superior to that with other *M* values. Compared with *M* = 2, the result of algorithm with *M* = 8 is relatively better on three of five test benchmarks. Therefore, the optimal setting of the parameter *M* can be *M* = 8 as general usage of the algorithm.

*(ii) Sensitivity in relation to T_Branch and T_Nmority in Level 2*. *T*_*Branch* and *T*_*Nmority* play vital roles in the population varying process, and there are critical correlations between them; thus, these two parameters are analyzed together. In this experiment, population number *M* is fixed to 8, and the values of *T*_*Branch* are varied as 5, 10, and 15 while the relevant *T*_*Nmority* is selected to be 0 or 5. From Table 5, it is clearly visible that the HARFO obtains best computation results on most test functions including *f*_11_, *f*_13_, *f*_14_, and *f*_15_ when *T*_*Branch*/*T*_*Nmority* are set as 10/5. Therefore, the optimal configuration of *T*_*Branch*/*T*_*Nmority* is 10/5 as general usage of the algorithm.

As the summary of this section, all suggested values of the control parameters can be listed as follows: *M* = 8, *T*_*Branch* = 10, and *T*_*Nmority* = 5. These values are determined by experiments where the correlative *T*_*Branch* and *T*_*Nmority* in level 2 are taken into consideration together, and the third one in level 1 is varied over an interval with a step size.

### 3.4. Computational Results

*(i) Comparative Results on 30-Dimensional Case*. HARFO is compared with ABC, PSO, and CCGA on ten 30-dimensional benchmarks *f*_1_–*f*_10_. On each benchmark, these algorithms are independently implemented 20 times and terminated when the number of function evaluations reaches 100,000 for each run. The statistical results in terms of mean and standard deviation of each benchmark over 20 runs are calculated and shown in Table 6. As revealed from experimental results in Table 6, HARFO generally shows relative outperformance for solving most test benchmarks, compared to CCGA and ABC, which obtain the second and third best rankings, respectively.

On *f*_1_, HARFO, CCGA, and ABC perform close to each other, relatively better than other algorithms. Specifically, CCGA does better than ABC, followed by CCGA. On *f*_2_, ARFO remarkably outperforms others as well as CCGA and ABC obtain close results, significantly better than other algorithms. For complex multimodal variable-separable *f*_3_, variable-separable *f*_4_, and nonseparable *f*_5_, HARFO performs slightly better than CCGA and ABC, furthermore significantly better than other algorithms. Particularly, on *f*_3_ and *f*_5_, the search performance order can be shown apparently as HARFO > CCGA > ABC > ARFO > PSO. On *f*_6_–*f*_10_, which are more complex shifted and rotated benchmarks, HARFO can obtain best performance in terms of mean, maximum, and minimum on most of five benchmarks including *f*_6_, *f*_8_, *f*_9_, and *f*_10_ and ARFO also outperforms other algorithms on *f*_7_. Apparently, HARFO shows significant improvement over other algorithms, especially ARFO.

*(ii) Comparative Results on 100-Dimensional Case*. In order to assess the scalability of our proposed algorithm, which is crucial for its applicability to real-world high-dimensional problems, the test benchmarks are extended to 100-dimensional problems as high-dimensional cases. The experimental results are given in Table 7. From Table 7, it is observed that HARFO significantly outperforms other algorithms almost all test functions except for *f*_2_ and *f*_7_. Particularly, compared to other algorithms, the solution accuracy of HARFO on shifted and rotated *f*_5_, *f*_9_, and *f*_10_ is increased by one order of magnitude. From the distinct difference between dimension = 20 and 100 results, it is clearly observed that with dimensionality increasing, the proposed algorithm exhibits its persistence and performs better.

Finally, the performance improvement obtained by our proposed algorithm can be generally explained: when other algorithms are trapped in the local optima, the HARFO can utilize the root-to-root communication mechanism to escape. By employing the hierarchical multipopulation coevolution, the complex task is decomposed into smaller-scale subproblems.

*(iii) Comparative Results on 30-Dimensional CEC 2014 Case*. Computation results in terms of means and stand deviations of the 20 runs obtained by six algorithms on ten 30-dimensional CEC 2014 benchmarks are given in Table 8, within which the best results among those algorithms are highlighted. From Table 8, the proposed HARFO performs significantly superior to its counterparts including CCGA and ABC on most of test benchmarks. Specifically, HARFO can do better than CCGA on *f*_11_, *f*_12_, *f*_14_, *f*_16_, *f*_17_, *f*_19_, and *f*_20_, followed by ABC and ARFO, PSO cannot obtain competitive results. On *f*_13_ and *f*_15_, ABC and CCGA perform significantly better than ARFO and slightly better than HARFO. PSO also obtains best result on *f*_18_. Furthermore, we can observe that although the shifted and rotated CEC 2014 benchmarks become more difficult to be handled compared with their classical counterparts and the computation results are not as satisfactory as those in the classical benchmarks, HARFO still performs more powerful than other algorithms on most test cases. This essentially indicates that HARFO has greater potential to cope with more complex problems.

## 4. Real-World Application for Image Segmentation

### 4.1. Otsu Criterion

The well-known Otsu criterion has been widely adopted to determine the optimal thresholds with desired characteristics through computing between-class variance [7, 8]. The original procedures of Otsu can be listed as below: at the beginning, a given image consisting of *N* pixels of gray levels falling into the range [0, *L* − 1] is taken into consideration. *h*(*i*) donates the pixel number of gray-level *i* and *P*(*i*) represents the probability of gray-level *i*.

Then, we have

(10) N=∑i=0L−1hi,Pi=hiN,for  0≤i≤L−1.

Hypothesize that *M* − 1 thresholds, namely, {*t*_1_, *t*_2_, …, *t*_*M*−1_}, are required to segment the given image into *M* classes: *C*_1_ for [0,…, 1], *C*_2_ for [*t*_1_ + 1,…, 1],…, *C*_*M*_ for [*t*_*M*−1_,…, *L*], the optimal thresholds {*t*_1_^*∗*^, *t*_2_^*∗*^, …, *t*_*M*−1_^*∗*^} selected by Otsu are described as

(11) t1∗,t2∗,…,tM−1∗=arg⁡max⁡σB2t1,t2,…,tM−1,0≤t1≤t2≤⋯≤tM−1≤L,

where

(12) σB2=∑i=1Mwi∗ui−uti∗2,wi=∑k∈Cik∗Pk,ui=∑k∈CiL=1k∗Pkwi,i=1,2,…,M.

Generally, (11) is employed as the fitness function for heuristic methods based procedure to be optimized. A close look into this equation will show that it is very similar to the expression for uniformity measure [40–43].

### 4.2. Experiment Setup

**Figure 4:**
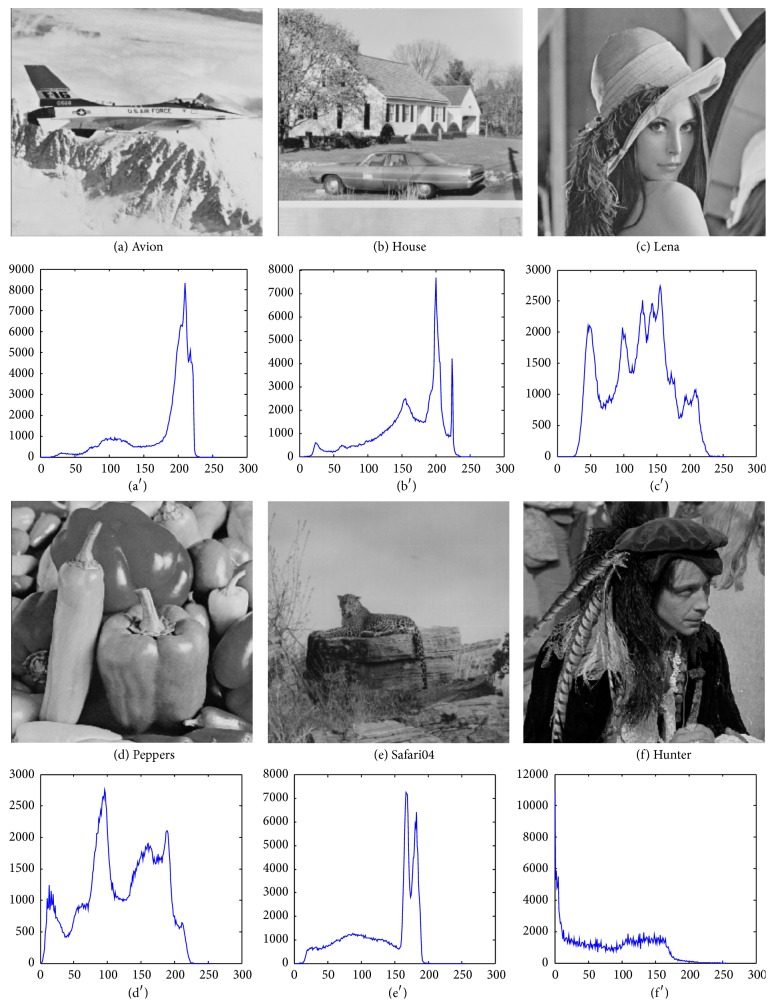
Test images and their histograms.

The image segmentation experiments by HARFO are conducted on a set of image datasets. These datasets consist of a variety of standard tested images widely employed in previous studies [42–47], including avion.ppm, house.ppm, lena.ppm, peppers.ppm, safari04.ppm, and hunter.pgm with pixels size of 512*∗*512 (available at http://decsai.ugr.es/cvg/dbimagenes/). Several state-of-the-art EA algorithms are selected for comprehensive comparison, namely, HARFO, ABC [20], ARFO [22], CCGA [39], and IDPSO [12]. Particularly, the IDPSO is an existing enhanced PSO variant with intermediate disturbance searching strategy recently proposed in [12] for image segmentation, which has gained satisfactory image segmentation *r* results. We will directly compare HARFO with existing computation results (i.e., for lena, peppers, and hunter) of IDPSO which have been reported in [12]. The parameters of HARFO, ABC, ARFO, PSO, and CCGA follow the optimal settings in Section 3.2. And the parameters of IDPSO are set the same as its original literature [12]. In this test, the proposed algorithm is to maximize the objective fitness within less computation time. The thresholds numbers *M* − 1 are set to 2, 3, 4, 5, 7, and 9. And Figure 4 presents test images and their histograms.

### 4.3. Experimental Results and Analysis

**Case 1 (segmentation results with *M* − 1 = 2,3, 4).**

Table 9 lists the objective values and mean computational time found by pure Otsu, which are partly reported in [12]. In practical real-time application, we hope that algorithms can keep a suitable balance of running time and high accuracy [48]. As shown in Table 9, due to the exhaustive search feature, Otsu needs to consume too long CPU time while achieving a satisfactory optimal thresholding. It can be seen from Table 10 that the proposed HARFO provides generally close results in terms of objective values and standard deviation compared with ABC and ARFO on some test cases, such as avion and lena. On lena, IDPSO and CCGA perform similarly, still a little worse than HARFO. At the same time considering the relevant results form Table 9, the proposed HARFO algorithm consumes less CPU time than its counterparts, which means that HARFO shows better efficiency. Compared with other algorithms, the HARFO has coevolution mechanism to perform better global search in higher-dimensional space.

**Case 2 (segmentation results with *M* − 1 = 5,7, 9).**

Table 11 gives computational results with *M* − 1 = 5, 7, and 9 in terms of average fitness and standard deviation of each algorithm. From Table 11, it is clearly observed that there are statistically significant differences between Cases 1 and 2 based on these segmentation algorithms, in aspects of both efficiency and stability. Due to the hybrid optimal strategies, HARFO exhibits obviously promising performance on this higher-dimensional segmentation case with *M* − 1 = 5, 7, and 9. Among these methods except HARFO, the ABC method also possesses relative powerful exploration ability due to usage of the scout bees operation. However, as shown in Table 11, as the number of segmentation thresholds increases, the results in terms of fitness values found by HARFO are significantly better than that of other methods, including the ABC algorithm. Generally, it can be concluded that HARFO performs better than other algorithms in this higher-dimensional scenario.

## 5. Conclusions

This paper proposes and develops a novel bionic optimization algorithm inspired by plant root growth mechanism to solve multilevel threshold image segmentation, namely, hybrid artificial root foraging optimizer (HARFO). Based on original single-colony ARFO, the potential of HARFO to improve the global search performance and keep diversity of population relies on the combination of the root-to-root communication and multipopulation cooperative mechanism. With root-to-root communication, information exchanging between individuals can be enhanced through different efficient topologies. With coevolution mechanism, the hierarchical spatial population driven by multipopulations is constructed to ensure that diversity of population is well kept.

The comparative experiments of HARFO in comparison with several classical population based algorithms are conducted on a set of 20-dimensional and 100-dimensional benchmarks. The experimental results validate the superiority of the proposed algorithm. Finally, the HARFO is employed to handle the image segmentation problems with multilevel threshold. Computational results achieved by this method on a suit of images dataset show that the proposed algorithm has significant potential to be a novel effective and efficient image processing approach. In our future work, we will focus on perfecting this novel optimization framework from the perspective of relevance theory.

## Figures and Tables

**Algorithm 1:**
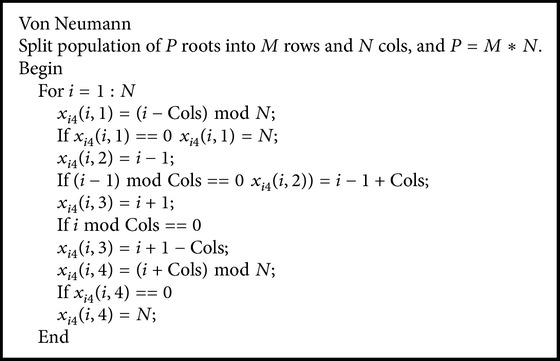
The pseudocode of Von Neumann.

**Algorithm 2:**
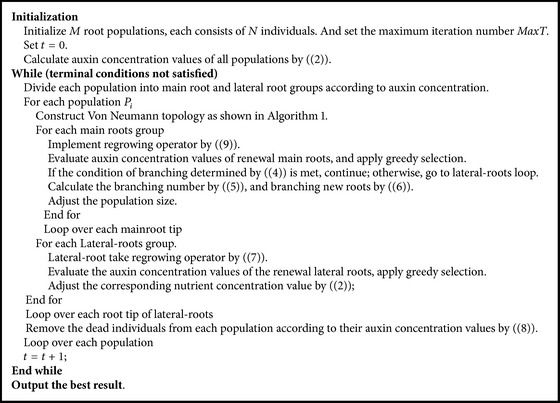
The pseudocode of HARFO.

**Table 1:** Parameters of basic benchmarks and CEC 2005 benchmarks (*x* ^*∗*^ is the optimal solution, *f*(*x* ^*∗*^) is the best values of function).

*f*	Functions	Dimensions	Initial range	*x* ^*∗*^	*f*(*x* ^*∗*^)
*f*_1_	Sphere function	20	[−100,100]^*D*^	[0,0,…, 0]	0
*f*_2_	Rosenbrock function	20	[−30,30]^*D*^	[1,1,…, 1]	0
*f*_3_	Rastrigrin function	20	[−5.12,5.12]^*D*^	[0,0,…, 0]	0
*f*_4_	Schwefel function	20	[−500, 500]^*D*^	[420.9867,…, 420.9867]	0
*f*_5_	Griewank function	20	[−600,600]^*D*^	[0,0,…, 0]	0
*f*_6_	Shifted Sphere Function	20	[−100, 100]^*D*^	[0,0,…, 0]	−450
*f*_7_	Shifted Rosenbrock's Function	20	[−100, 100]^*D*^	[0,0,…, 0]	390
*f*_8_	Shifted Schwefel's Problem	20	[−100, 100]^*D*^	[0,0,…, 0]	−450
*f*_9_	Shifted Rotated Griewank's Function without Bounds	20	No bounds	[0,0,…, 0]	−180
*f*_10_	Shifted Rastrigin's Function	20	[−5,5]^*D*^	[0,0,…, 0]	−330

**Table 2:** Parameters of CEC 2014 test functions (*x* ^*∗*^ is the optimal solution; *f*(*x* ^*∗*^) is the best values of function; and *O*_*i*_ is the shifted global optimum defined in “shift_data_x.txt,” which is randomly distributed in [−80,80]^*D*^).

*f*	Functions	Dimensions	Initial range	*x* ^*∗*^	*f*(*x* ^*∗*^)
*f*_11_	Rotated High Conditioned Elliptic Function	30	[−100,100]^*D*^	*O*_1_	100
*f*_12_	Rotated Bent Cigar Function	30	[−100,100]^*D*^	*O*_2_	200
*f*_13_	Rotated Discus Function	30	[−100,100]^*D*^	*O*_3_	300
*f*_14_	Shifted and Rotated Rosenbrock's Function	30	[−100,100]^*D*^	*O*_4_	400
*f*_15_	Shifted and Rotated Ackley's Function	30	[−100,100]^*D*^	*O*_5_	500
*f*_16_	Shifted and Rotated Weierstrass Function	30	[−100,100]^*D*^	*O*_6_	600
*f*_17_	Shifted and Rotated Griewank's Function	30	[−100,100]^*D*^	*O*_7_	700
*f*_18_	Shifted Rastrigin's Function	30	[−100,100]^*D*^	*O*_8_	800
*f*_19_	Shifted and Rotated Rastrigin's Function	30	[−100,100]^*D*^	*O*_9_	900
*f*_20_	Shifted Schwefel's Function	30	[−100,100]^*D*^	*O*_10_	1000

**Table 3:** Parameters of HARFO and ARFO for optimization.

HARFO	
The number of initial population	20
The maximum number of population	100
*T_Branch*	10
*T_Nmority*	5
*S*_*max*_	4
*S*_*min*_	1
Population number	8
The number of initial population	4
The maximum number of single population	50
BranchG	10
Nmority	5
*S*_*max*_	4
*S*_*min*_	1

**Table 4:** Results obtained by HARFO with different *population number*.

*M*		2	5	8	11	14	17
*f*_11_	Mean	**1.9663E** + **01**	3.0644*E* + 01	2.0535*E* + 01	3.4134*E* + 01	3.7901*E* + 01	3.4393*E* + 01
	Std	6.5353*E* − 01	7.3232*E* − 01	**4.9023E** − **01**	8.1423*E* − 01	9.0482*E* − 01	8.2108*E* − 01

*f*_12_	Mean	6.5523*E* + 01	7.3533*E* + 01	**4.9234E** + **01**	8.1788*E* + 01	9.0872*E* + 01	8.2462*E* + 01
	Std	1.1212*E* + 02	1.2511*E* + 02	**8.4181E** + **01**	1.3922*E* + 02	1.5537*E* + 02	1.4099*E* + 02

*f*_13_	Mean	**3.1865E** + **01**	3.5876*E* + 01	3.7405*E* + 01	6.2144*E* + 01	6.9037*E* + 01	6.2648*E* + 01
	Std	**1.0089E** + **01**	1.9254*E* + 01	1.2886*E* + 01	2.1429*E* + 01	2.3784*E* + 01	2.1583*E* + 01

*f*_14_	Mean	3.7411*E* + 01	4.1955*E* + 01	**2.8088E** + **01**	4.6603*E* + 01	5.1842*E* + 01	4.7044*E* + 01
	Std	9.6699*E* − 01	1.0881*E* + 00	**7.2544E** − **01**	1.2127*E* + 00	1.3389*E* + 00	1.2150*E* + 00

*f*_15_	Mean	7.9732*E* + 01	8.9323*E* + 01	**5.9839E** + **01**	9.9226*E* + 01	1.1045*E* + 02	1.0022*E* + 02
	Std	3.3109*E* + 01	3.7102*E* + 01	**2.4897E** + **01**	4.1900*E* + 01	4.5952*E* + 01	4.1699*E* + 01

**Table 5:** Results obtained by HARFO with different *T_Branch *and *T_Nmority*.

*T_Branch/T_Nmority*		5/0	10/0	15/0	5/5	10/5	15/5
*f*_11_	Mean	2.9494*E* + 01	2.9835*E* − 01	1.7934*E* + 01	2.0332*E* + 01	**1.6815E + 01**	3.0914*E* + 02
	Std	6.0931*E* − 01	3.9945	6.1453*E* − 01	5.0094*E* − 01	**4.0143E − 01**	9.0093

*f*_12_	Mean	4.5534*E* + 02	2.4346*E* + 03	8.6729*E* − 01	2.4621*E* + 02	4.0316*E* + 01	**3.4344E + 01**
	Std	2.3424*E* + 02	1.5529*E* + 02	3.4031*E* + 01	1.6436*E* + 02	6.8932*E* + 01	**5.6987**

*f*_13_	Mean	3.0352*E* + 01	6.3321*E* + 01	3.6239*E* + 01	3.1234*E* + 02	**3.0629E + 01**	1.0342*E* + 02
	Std	4.0945*E* + 01	8.0345*E* + 01	2.9023*E* + 01	9.0945*E* + 01	**1.0552E + 01**	9.0934*E* + 01

*f*_14_	Mean	4.0333*E* + 01	1.4452*E* + 02	4.8845*E* + 02	2.0340*E* + 02	**2.3000E + 01**	**2.4136E + 00**
	Std	7.9834	6.8554	5.2231*E* + 01	2.4442*E* + 01	**5.9403E − 01**	5.9453*E* − 01

*f*_15_	Mean	1.0934*E* + 02	5.5423*E* + 01	4.9000*E* + 01	2.2454*E* + 02	**4.9000E + 01**	1.0043*E* + 02
	Std	4.2213*E* + 02	4.7775*E* + 01	6.0003*E* + 01	8.8896*E* + 01	**2.0387E + 01**	3.2009*E* + 01

**Table 6:** Comparison of results with 30 dimensions obtained by each algorithm (*f*(*x*) − *f*(*x* ^*∗*^)).

Func.		HARFO	ABC	ARFO	PSO	CCGA
*f*_1_	Mean	4.2817*E* − 13	1.3230*E* − 20	8.4537*E* − 03	6.4050*E* − 01	**1.2942E − 20**
	Std	5.8831*E* − 13	**4.3300E − 20**	6.6176*E* − 03	2.2735*E* − 01	4.2357*E* − 20

*f*_2_	Mean	**7.2733E − 01**	3.9681*E* + 00	6.9380*E* + 01	2.6402*E* + 02	3.8817*E* + 00
	Std	1.5903*E* + 00	3.7709*E* + 00	**1.3271E + 00**	1.4057*E* + 02	3.6888

*f*_3_	Mean	**2.1575E − 13**	4.9539*E* + 01	7.5788*E* + 01	1.3201*E* + 02	4.8976*E* + 01
	Std	**1.2233E − 12**	1.3063*E* + 01	1.1519*E* + 00	3.7770*E* + 02	1.2903*E* + 01

*f*_4_	Mean	7.6514*E* − 04	**2.8590E − 04**	4.4610*E* + 03	2.9702*E* + 02	**2.8094E − 04**
	Std	6.3836*E* − 04	2.7604*E* − 04	2.2428*E* + 02	2.1757*E* + 02	**1.5404E − 04**

*f*_5_	Mean	**5.0157E − 03**	8.3921*E* − 02	9.2917*E* − 01	3.9481*E* + 00	8.2891*E* − 02
	Std	4.9156*E* − 03	7.3446*E* − 02	2.8713*E* − 01	**2.6640E − 03**	7.2544*E* − 02

*f*_6_	Mean	**4.0543E − 14**	7.5624*E* − 14	6.3095*E* + 02	9.4363*E* + 01	7.4696*E* − 14
	Std	3.4998*E* − 14	**3.0806E − 14**	7.8376*E* + 02	3.9237*E* + 01	6.6428*E* − 14

*f*_7_	Mean	8.4784*E* + 00	2.4324*E* + 01	**1.4680E + 00**	7.0365*E* + 06	2.4025*E* + 01
	Std	**1.2762E + 00**	7.1995*E* + 01	2.0352*E* + 00	2.3168*E* + 07	7.1111*E* + 01

*f*_8_	Mean	**1.9573E + 02**	9.0699*E* + 02	1.9471*E* + 02	2.0902*E* + 04	8.9575*E* + 02
	Std	4.6811*E* + 02	5.8412*E* + 02	**1.5947E + 01**	4.8404*E* + 03	5.7698*E* + 02

*f*_9_	Mean	**1.6015E + 03**	2.0949*E* + 03	5.2497*E* + 03	2.5180*E* + 03	2.0664*E* + 03
	Std	6.9952*E* − 01	**7.4802E − 13**	5.2374*E* + 02	3.8381*E* + 02	7.6612*E* − 13

*f*_10_	Mean	**6.8062E + 00**	5.9028*E* + 01	3.4875*E* + 02	6.6128*E* + 01	5.8311*E* + 01
	Std	6.4614*E* − 01	1.7745*E* − 01	6.5806*E* + 01	5.7449*E* + 00	**1.7554E − 01**

**Table 7:** Comparison of results with 100 dimensions obtained by each algorithm (*f*(*x*) − *f*(*x* ^*∗*^)).

Func.		HARFO	ABC	ARFO	PSO	CCGA
*f*_1_	Mean	1.2867*E* − 03	2.5048*E* − 03	4.3104*E* − 02	4.5449*E* + 02	**1.2558E − 02**
	Std	6.2167*E* − 03	5.0736*E* − 03	7.8429*E* − 03	1.1368*E* + 02	**5.7436E − 03**

*f*_2_	Mean	2.3158*E* + 02	6.2667*E* + 02	6.0868*E* + 02	1.0874*E* + 04	**9.8880E + 01**
	Std	**2.9080E + 01**	6.8733*E* + 02	3.3206*E* + 01	6.7649*E* + 04	3.2121*E* + 01

*f*_3_	Mean	**6.7884E + 02**	1.4622*E* + 02	9.1064*E* + 02	5.6672*E* + 03	8.8089*E* + 02
	Std	1.3927*E* + 02	1.8944*E* + 01	**6.6473E + 01**	7.2860*E* + 02	6.7302*E* + 01

*f*_4_	Mean	**1.7000E + 01**	5.1011*E* + 02	**1.7000E + 01**	**1.7000E + 01**	1.8874*E* + 01
	Std	4.0850*E* + 00	1.2089*E* + 02	**2.3858E + 00**	7.2467*E* + 00	2.6488*E* + 00

*f*_5_	Mean	1.7451*E* − 01	5.4011*E* + 01	1.6357*E* + 01	1.5808*E* + 03	**1.7160E − 01**
	Std	2.1547*E* − 01	2.7978*E* + 01	4.4982*E* + 00	3.2400*E* + 02	**1.9940E − 01**

*f*_6_	Mean	**6.4416E + 03**	7.2900*E* + 04	9.8642*E* + 03	2.3474*E* + 05	9.5026*E* + 03
	Std	1.1540*E* + 03	2.5311*E* + 03	**1.0969E + 03**	4.0396*E* + 04	1.1567*E* + 03

*f*_7_	Mean	3.3781*E* + 10	2.8028*E* + 10	**1.1095E + 09**	6.8711*E* + 09	1.1688*E* + 09
	Std	1.1606*E* + 10	8.0320*E* + 09	4.6126*E* + 08	**1.9879E + 08**	4.4435*E* + 08

*f*_8_	Mean	**1.2057E + 05**	3.5396*E* + 05	1.2118*E* + 05	4.5362*E* + 05	1.3464*E* + 05
	Std	1.7588*E* + 04	9.1560*E* + 04	**4.1061E + 03**	3.1074*E* + 04	4.5623*E* + 03

*f*_9_	Mean	**1.5149E + 03**	1.8168*E* + 04	3.5279*E* + 04	2.8477*E* + 04	3.9199*E* + 04
	Std	**1.0312E + 02**	2.7420*E* + 03	1.3535*E* + 03	8.9127*E* + 02	1.5039*E* + 03

*f*_10_	Mean	**2.2175E + 02**	6.5384*E* + 03	1.1434*E* + 03	1.8442*E* + 03	1.2704*E* + 03
	Std	3.7382*E* + 00	**3.6678E + 00**	1.2423*E* + 02	2.2994*E* + 02	1.3803*E* + 02

**Table 8:** Comparison of results with 30 dimensions obtained by each algorithm (*f*(*x*) − *f*(*x* ^*∗*^)).

Func.		HARFO	ABC	ARFO	PSO	CCGA
*f*_11_	Mean	**1.6815E + 01**	1.9799*E* + 07	1.2573*E* + 07	2.9358*E* + 08	1.9563*E* + 02
	Std	**3.9994E − 01**	6.7129*E* + 06	1.0473*E* + 07	2.1609*E* + 08	6.6330*E* + 02

*f*_12_	Mean	**3.1528E + 01**	7.5904*E* + 02	1.0104*E* + 08	7.0153*E* + 08	7.5001*E* + 02
	Std	**2.4023E + 01**	7.5572*E* + 02	2.3503*E* + 08	2.8266*E* + 08	7.4673*E* + 02

*f*_13_	Mean	2.8969*E* + 01	**1.3000E + 01**	1.5265*E* + 02	4.5742*E* + 02	**1.3000E + 01**
	Std	1.5290*E* + 00	**2.5348E + 01**	5.0791*E* + 01	1.5825*E* + 02	2.8136*E* + 01

*f*_14_	Mean	**2.2457E + 01**	2.4494*E* + 01	2.4296*E* + 01	2.5464*E* + 01	2.7188*E* + 01
	Std	4.8265*E* − 01	4.8600*E* − 02	**1.2623E − 02**	7.6957*E* − 02	5.3946*E* − 02

*f*_15_	Mean	4.2697*E* + 01	**1.9000E + 01**	3.6291*E* + 01	5.0531*E* + 01	**1.9000E + 01**
	Std	8.1373*E* + 00	1.7665*E* + 00	3.1039*E* + 00	2.2499*E* + 00	**1.0608E + 00**

*f*_16_	Mean	**3.1060E − 03**	1.3200*E* − 03	5.6546*E* + 00	1.2402*E* + 02	1.4652*E* − 03
	Std	**1.5530E − 03**	3.8400*E* − 03	7.0040*E* + 00	3.0668*E* + 01	4.4330*E* − 03

*f*_17_	Mean	**6.1585E − 06**	5.1208*E* + 02	1.5884*E* + 02	3.1284*E *+ 02	5.9117*E* + 02
	Std	**3.3153E − 05**	1.1126*E* + 02	3.0743*E* + 01	4.1798*E* + 01	1.2844*E* + 02

*f*_18_	Mean	2.1913*E* + 03	6.1602*E* + 06	3.5613*E* + 05	**2.0148E + 03**	7.1116*E* + 06
	Std	4.7034*E* + 02	3.4051*E* + 06	7.1417*E* + 05	**3.4323E + 02**	3.9310*E* + 06

*f*_19_	Mean	**4.7272E + 02**	1.6622*E* + 04	8.5742*E* + 02	7.3078*E* + 06	1.5327*E* + 04
	Std	2.2216*E* + 02	1.4328*E* + 03	**1.2094E + 02**	5.5582*E* + 06	1.3212*E* + 03

*f*_20_	Mean	**2.4618E + 02**	2.7362*E* + 02	2.9471*E* + 02	4.7931*E* + 02	2.5231*E* + 02
	Std	**1.2685E + 01**	1.6578*E* + 02	9.1791*E* + 02	2.9374*E* + 01	1.5287*E* + 02

**Table 9:** Objective values and thresholds by the Otsu method.

Image	*M* − 1 = 2		*M* − 1 = 3		*M* − 1 = 4	
	Objective values	Optimal thresholds	Objective values	Optimal thresholds	Objective values	Optimal thresholds
Avion	3.493*E* + 3	113, 173	3.13*E* + 4	93, 145, 191	3.45*E* + 4	84, 129, 172, 203
House	2.24*E* + 3	107, 173	2.42*E* + 4	84, 137, 181	2.86*E* + 4	71, 118, 153, 186
Lena	9.34*E* + 3	134, 165,	1.13*E* + 4	121, 151, 176	1.26*E* + 4	111, 140, 158, 180
Peppers	9.35*E* + 3	134, 176	1.13*E* + 4	113, 158, 184	1.25*E* + 4	103, 140, 167, 189
Safari04	2.53*E* + 3	82, 141	2.33*E* + 4	65, 107, 151	2.43*E* + 4	55, 88, 120, 156
Hunter	5.45*E* + 3	102, 146	5.45*E* + 3	86, 129, 155	9.75*E* + 3	69, 112, 137, 158
Mean CPU time	2.1472		175.776		7945.325	

**Table 10:** Objective values and standard deviation by heuristic methods on Otsu algorithm.

Image	*M* − 1	Objective values (standard deviation)				
		HARFO	ABC	ARFO	CCGA	IDPSO
Avion	2	4.3909*E* + 04	3.8752*E* + 04	3.8870*E* + 04	3.902*E* + 04	3.948*E* + 04
		3.8166*E* − 01	8.7275*E* − 12	1.8550*E* − 02	8.642*E* − 02	1.594*E* − 01
	3	4.4003*E* + 04	3.8839*E* + 04	3.8948*E* + 04	4.034*E* + 04	4.013*E* + 04
		2.0072*E* − 01	2.1530*E* − 01	1.2917*E* − 01	4.442*E* − 01	5.238*E* − 01
	4	4.4031*E* + 04	3.8889*E* + 04	4.4001*E* + 04	4.441*E* + 04	4.111*E* + 04
		5.9884*E* − 01	5.3222*E* − 01	9.4322*E* − 01	1.542*E* − 01	2.442*E* − 01

House	2	3.6206*E* + 04	3.1941*E* + 04	3.2069*E* + 04	3.423*E* + 04	3.551*E* + 04
		3.7387*E* − 02	0.0000*E* + 00	7.0560*E* − 02	2.194*E* − 01	5.422*E* − 04
	3	3.6371*E* + 04	3.2082*E* + 04	3.2209*E* + 04	3.441*E* + 04	3.532*E* + 04
		3.5218*E* − 02	4.2787*E* − 02	2.2613*E* − 01	5.213*E* − 01	7.522*E* − 03
	4	3.6430*E* + 04	3.2137*E* + 04	3.2332*E* + 04	3.424*E* + 04	3.575*E* + 04
		1.0542*E* + 00	5.4974*E* − 01	7.3052*E* − 01	2.113*E* + 00	5.094*E* − 02

Lena	2	2.2663*E* + 04	1.3928*E* + 04	1.0034*E* + 04	2.132*E* + 03	9.3449*E*3
		1.0934*E* − 02	5.0944*E* − 12	5.4093*E* − 02	2.422*E* − 01	5.46*E* − 12
	3	2.4983*E* + 04	2.0233*E* + 04	2.1033*E* + 04	2.142*E* + 04	1.1334*E*4
		8.0934*E* − 03	7.42343*E* − 02	4.4421*E* − 01	2.499*E* − 02	9.095*E* − 12
	4	2.2742*E* + 04	2.0003*E* + 04	1.9999*E* + 04	2.042*E* + 04	1.2558*E*4
		2.6544*E* − 01	6.42245*E* − 01	5.4226	5.453	4.336*E* − 1

Peppers	2	1.0340*E* + 04	1.9533*E* + 04	2.0344*E* + 04	9.924*E* + 03	9.3515*E*3
		4.5333*E* − 12	5.34337*E* − 12	7.5652*E* − 02	5.424*E* − 01	1.27*E* − 11
	3	1.1322*E* + 04	1.0125*E* + 04	1.1333*E* + 04	1.153*E* + 04	1.1269*E*4
		1.4422*E* + 00	2.5566*E* − 02	2.4223*E* − 01	5.5301	5.46*E* − 12
	4	1.9834*E* + 04	1.9593*E* + 04	1.9818*E* + 04	1.993*E* + 04	1.2525*E*4
		9.0452*E* − 11	9.7863*E* − 02	1.9887	4.522*E* − 01	5.45*E* − 12

Safari04	2	2.5866*E* + 04	2.2766*E* + 04	2.2984*E* + 04	2.414*E* + 04	2.363*E* + 04
		5.6183*E* − 01	0.0000*E* + 00	4.7684*E* − 02	5.252*E* − 01	5.414*E* − 02
	3	2.5954*E* + 04	2.2865*E* + 04	2.3015*E* + 04	2.422*E* + 04	2.309*E* + 04
		9.5100*E* − 01	3.5163*E* − 02	2.0405*E* − 01	7.532*E* − 01	5.522*E* − 01
	4	2.6005*E* + 04	2.2903*E* + 04	2.3122*E* + 04	2.443*E* + 04	2.442*E* + 04
		7.0754*E* − 01	1.0771*E* + 00	1.8869*E* − 01	2.0934	5.720*E* − 02

Hunter	2	2.2311*E* + 04	1.0378*E* + 04	1.0389*E* + 04	5.042*E* + 02	5.4491*E*3
		0	0	2.0462*E* − 01	5.642*E* − 01	0
	3	6.5233*E* + 03	3.0422*E* + 03	6.5222*E* + 03	6.043*E* + 03	6.4260*E*3
		2.4122	8.4544	5.4223*E* − 01	5.3252	1.774*E* − 1
	4	6.4522*E* + 04	1.1444*E* + 04	6.8632*E* + 03	1.214*E* + 04	6.9721*E*3
		1.0222	5.7733	5.0530	3.534	1.4463

**Table 11:** Objective value and standard deviation by the compared population based methods on Otsu algorithm.

Image	*M* − 1	Objective values (standard deviation)				
		HARFO	ABC	ARFO	CCGA	IDPSO
Avion	5	4.2940*E* + 04	4.0947*E* + 04	4.2721*E* + 04	4.266*E* + 04	4.165*E* + 04
		6.6839*E* − 02	1.5094*E* + 00	1.2493*E* + 00	3.133*E* − 02	4.9845*E* − 02
	7	4.2852*E* + 04	4.0972*E* + 04	4.2735*E* + 04	4.266*E* + 04	4.243*E* + 04
		3.0811*E* − 02	3.8286*E* + 00	1.6504*E* + 00	4.531*E* − 01	5.325*E* − 01
	9	4.2857*E* + 04	4.0986*E* + 04	4.2639*E* + 04	4.275*E* + 04	4.293*E* + 04
		1.3309*E* − 01	2.6222*E* + 00	5.4868*E* − 01	2.853*E* − 01	5.535*E* − 01

House	5	3.5543*E* + 04	3.3854*E* + 04	3.5364*E* + 04	3.443*E* + 04	3.468*E* + 04
		5.3886*E* − 01	1.8866*E* + 00	5.8096*E* − 01	2.284*E* − 01	8.653*E* − 02
	7	3.5558*E* + 04	3.3895*E* + 04	3.5360*E* + 04	3.512*E* + 04	3.574*E* + 04
		1.0541*E* − 01	5.4665*E* + 00	1.7216*E* + 00	5.543*E* − 01	5.524*E* − 01
	9	3.5610*E* + 04	3.3919*E* + 04	3.5263*E* + 04	3.521*E* + 04	3.341*E* + 04
		1.4416*E* − 01	3.8422*E* + 00	1.4566*E* + 00	2.842	6.751*E* − 01

Lena	5	1.2141*E* + 04	1.1077*E* + 04	1.1890*E* + 04	1.340*E* + 04	1.3399*E*4
		1.1142*E* − 02	2.3456*E* + 00	6.9755*E* − 01	2.434*E* − 01	2.01*E* − 2
	7	1.2232*E* + 04	1.1144*E* + 04	1.1942*E* + 04	1.135*E* + 04	1.4418*E*4
		1.2534*E* − 01	5.0432*E* + 00	8.9221*E* − 01	2.743	4.2087
	9	1.2154*E* + 04	1.1424*E* + 04	1.2012*E* + 04	1.133*E* + 04	1.4984*E*4
		1.7543*E* − 01	3.8324*E* + 00	1.7423*E* + 00	3.326	6.457

Peppers	5	2.1900*E* + 04	2.0668*E* + 04	2.1587*E* + 04	2.202*E* + 04	1.3366*E*4
		5.0443*E* − 02	1.7541*E* + 00	2.0475*E* + 00	2.1408*E* − 01	5.019*E* − 1
	7	2.1717*E* + 04	2.0710*E* + 04	2.1531*E* + 04	1.522*E* + 04	1.4293*E*4
		1.3917*E* − 01	2.8835*E* + 00	2.6158*E* + 00	2.244	1.1924*E* + 1
	9	2.1743*E* + 04	2.0725*E* + 04	2.1645*E* + 04	1.662*E* + 04	1.4792*E*4
		2.3165*E* − 01	2.8313	2.3470	6.354	9.3027

Safari04	5	2.5363*E* + 04	2.4130*E* + 04	2.5172*E* + 04	2.319*E* + 04	2.516*E* + 04
		1.1934*E* − 01	6.1834*E* + 00	1.6155*E* + 00	1.542*E* − 01	2.526*E* + 00
	7	2.5449*E* + 04	2.4154*E* + 04	2.5225*E* + 04	2.524*E* + 04	2.413*E* + 04
		1.3396*E* − 01	4.8186*E* + 00	1.2653*E* + 00	4.563*E* − 01	6.224*E* − 01
	9	2.5472*E* + 04	2.4165*E* + 04	2.5062*E* + 04	2.401*E* + 04	2.446*E* + 04
		1.0982*E* − 01	1.9172*E* + 00	5.8804*E* − 01	2.536	5.514*E* − 01

Hunter	5	1.1723*E* + 04	1.1205*E* + 04	1.1614*E* + 04	1.113*E* + 04	7.350*E*3
		9.4823*E* − 02	4.7343*E* + 00	1.6177*E* + 00	4.562*E* − 01	5.1693
	7	1.1774*E* + 04	1.1242*E* + 04	1.1668*E* + 04	1.102*E* + 04	7.752*E*3
		2.0734*E* − 01	4.3856*E* + 00	1.8600*E* + 00	2.326	9.7143
	9	1.1805*E* + 04	1.1260*E* + 04	1.1695*E* + 04	1.101*E* + 04	7.974*E*3
		1.5995*E* − 01	3.6223*E* + 00	4.0659*E* + 00	2.563	1.620*E* + 1
